# Manifestation of Cytomegalovirus-Associated Gastritis and Colitis With Immunosuppression and Review of Literature

**DOI:** 10.1155/crdi/1143576

**Published:** 2025-01-16

**Authors:** Ahmad Mirza, John Erikson Yap, Rajan Kapoor, Imran Gani

**Affiliations:** Department of Transplant, Medical College of Georgia, Augusta University, 1120 15th Street, AD 3401, Augusta, Georgia

**Keywords:** colitis, cytomegalovirus, immunosuppression, kidney transplant

## Abstract

Cytomegalovirus (CMV) infection in immunocompromised patients can cause significant morbidity and mortality. Early recognition and treatment helps to improve outcome. We present a case of postrenal transplant CMV infection causing both upper and lower gastrointestinal infection and symptoms. Patient developed significant co-morbidity which required multiple hospital admissions and therapeutic interventions.

## 1. Introduction

The increased risk of infection in kidney transplant patients is largely due to antirejection immunosuppressive regimens. Early infections post-transplant are linked to higher rates of graft rejection and all-cause mortality [1–3]. Cytomegalovirus (CMV) infection is a serious complication in postkidney transplant patient population, arising commonly from reactivation or de novo transmission in kidney transplant recipients. This may occur when a seronegative recipient (R−) patient receives a graft from a seropositive donor (D+). Seropositive recipient (R+) cases are also observed, albeit at lower rates, with an insignificant disparity in disease severity between D + R + and D + R − matches [4]. Transmission of active CMV infection from the transplanted kidney to the recipient is another cause of CMV disease. Clinical manifestations of CMV infections are variable and can range from asymptomatic presentation to severe and life-threatening complications.

This case addresses the manifestation of CMV colitis and CMV duodenitis in an elderly African American patient, with focus on diagnosis, disease progression, and interdisciplinary management.

## 2. Case Report

A 70-year-old African American patient with history of hypertension (HTN), diabetes mellitus (DM), and end-stage renal disease (ESRD) due to hypertensive nephrosclerosis underwent right iliac fossa deceased donor kidney transplant (DDKT) without complication from a 60-year-old donor. Both the virtual (computed generated comparison to identify pre-formed antibodies in the recipient against the donor antigen profile) and physical cross-matching (mixing of donor and recipient blood to establish acceptability of organ when no hemolysis occurs under the microscope) were performed prior to transplantation. Induction immunosuppressive therapy was started with intravenous thymoglobulin (1.5 mg/kg). The thymoglobulin was administered during transplant procedure and post-op Days 1, 2, and 3. Oral Immunosuppressive maintenance therapy was initiated with oral tacrolimus (0.1 mg/kg, twice daily), mycophenolate mofetil (750 mg twice daily), and prednisone (5 mg daily). Immediate patient's postoperative recovery was complicated by an episode of acute respiratory distress due to perioperative aspiration on post-op day (POD) 3. Patient became tachycardic (HR 142/min), tachypneic (26/min), and decreased oxygen saturations (88%). Patient was managed with high flow nasal canula and admitted to the surgical intensive care unit. Chest x-ray film was diagnostic for consolidation in the right lower lobe. Patient's white cell count was elevated (16,000 cells cells per microliter (cells/μL). Clinical diagnosis of aspiration pneumonia was confirmed. Patient was administered a 5 days course of intravenous augmentin (1.2 g IV twice daily) and metronidazone (500 mg IV three times daily). After improvement patient was converted to oral augmentin (625 mg three times daily) to complete a full seven days antibiotics course. Patient underwent aggressive physiotherapy. After making good recovery patient was discharged on POD 8.

Also, immediately post-transplant appropriate prophylaxis against opportunistic infections was initiated with nystatin (100,000 unit/mL, oral fungal infection prevention), trimethoprim/sulfamethoxazole (80/400 mg daily, prophylaxis against Pneumocystis carinii pneumonia), and oral valganciclovir (450 mg daily, for CMV prophylaxis). The kidney donor was CMV negative and recipient was CMV IgG positive, indicating previous exposure. Dose adjustments were made as guided by patient's creatinine clearance (At 6 months prophylaxis was stopped after completing the standard prophylaxis protocol).

**Figure 1:**
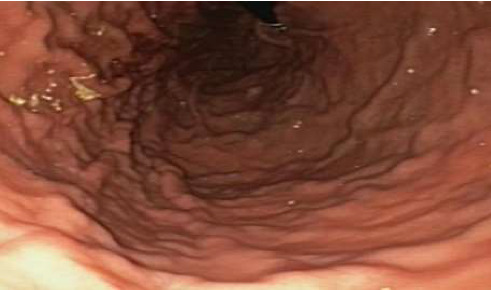
Normal looking gastric mucosa on retroflexion of endoscope.

**Figure 2:**
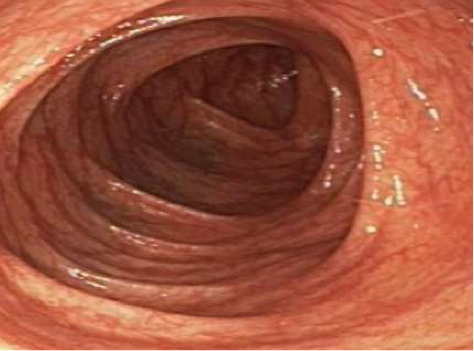
A normal looking transverse colonic mucosa.

**Figure 3:**
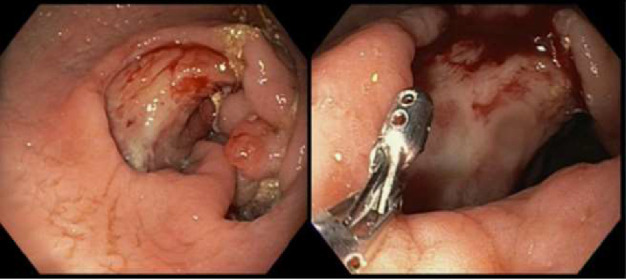
A large deep 40 × 50 mm clean-based ulcer seen in antrum. Multiple biopsies performed at the edge and centre of the ulcer.

**Figure 4:**
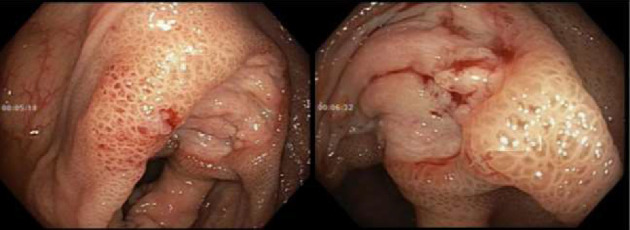
A large 30 × 40 mm clean-based ulcer seen in the ascending colon near the ileocecal valve.

**Figure 5:**
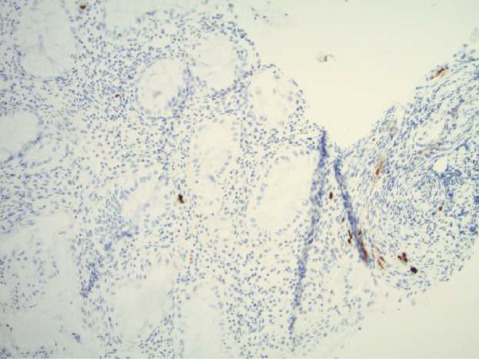
CMV immunostain showing positive nuclear and cytoplasmic staining in colonic mucosa.

**Figure 6:**
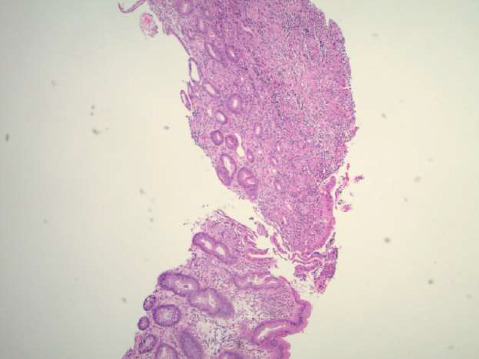
Low power view (4x) of H&E stained slide of colon biopsy showing ulceration and granulation tissue.

**Figure 7:**
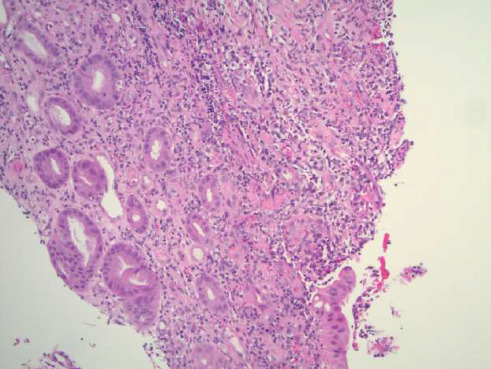
High power (20x) view showing ulceration, inflammation, and granulation tissue of the colonic mucosa.

Initially patient was followed in the transplant medicine clinic every week immediately after kidney transplant up to 6 weeks. Thereafter since patient was making satisfactory progress clinic review was completed every 8 weeks. Patient was progressing well and free of dialysis. At 18 months following transplantation patient developed symptomatic anemia and was admitted with low hemoglobin (Hb-7.6 g/dL) and associated shortness of breath (heart rate 76/minute, respiratory rate 14/min and oxygen saturations 94% room air). Patient was also found to have positive fecal occult blood test. Therefore inpatient esophagogastroduodenoscopy (EGD) and colonoscopy were performed as standard diagnostic workup for new onset of iron deficiency anemia (IDA). The endoscopic and colonoscopy findings from noninvolved areas are described in Figures 1 and 2. The endoscopy also revealed a single large, deep clean base ulcer in the antrum (4 × 5 cm) and pylorus (0.6 × 0.5 cm) (Figure 3). On colonoscopy a single deep ulcer was identified in the ascending colon (3 × 4 cm) (Figure 4). Multiple biopsies were performed, with negative findings for the upper gastrointestinal (GI) lesions, but staining of colonic ulcerative tissue returned positive for CMV (Figures 5, 6 and 7). The results from quantitative CMV polymerase chain reaction (PCR) reported 263 IU/mL (normal < 54 IU(International Units)/mL, Artus CMV RGQ MDx Kit is an in vitro nucleic acid amplification test for the quantitation of human CMV DNA in human plasma). The combination of immunohistochemistry and positive viral load on PCR confirmed diagnosis of tissue invasive CMV infection in an immunocompromised patient. Patient was started on intravenous ganciclovir (2.5 mg/kg) once daily based on creatinine clearance. Mycophenolate mofetil was stopped at this stage. After three intravenous doses patient felt clinical improvement and was started on oral valganciclovir (450 mg) twice daily and discharged home in a stable condition.

After one month of outpatient follow-up, patient was re-admitted with persistent diarrhea, abdominal pain, and a 10-pound weight loss. Because of patients' inability to take medicines orally, IV ganciclovir was reinitiated. Following initial intravenous treatment, patient made considerable improvement and was started again on oral valganciclovir (450 mg) twice daily and discharged.

The following month, patient returned to the emergency department with persistent hematemesis and bloody stools. EGD and biopsy were performed to rule out a source of upper GI bleeding. It revealed the same findings as the previous EGD with a single, large ulcer now spanning the antrum and pylorus. EGD findings were consistent with chronic fibrinopurulent gastritis and pathological reports returned negative for malignancy and *H. pylori*. The serum CMV viral copy number was no longer detectable by PCR. A week after discharge, patient was again readmitted with bloody stools and assessed by interventional radiology for persistent GI bleeding. Gastroenterology teams were also consulted, however, because of the location and size of the ulcer, endoscopic intervention was considered futile. Therefore, patient underwent mesenteric angiogram with embolization of the right gastroepiploic and gastroduodenal arteries without complications.

The patient's CMV management remained successful. However, patient returned to the emergency department 2 weeks after embolization due to concerns related to asymptomatic urinary tract infection (UTI). The urine cultures grew vancomycin-resistant enterococcus (VRE) and underwent treatment with linezolid (600 mg twice daily) for 2 weeks with repeat cultures showing resolution of VRE and no UTI symptoms.

## 3. Discussion

This case presents the management of an elderly postkidney transplant patient diagnosed with both CMV colitis and duodenitis. This most likely resulted from immunocompromised state of the recipient. The immunosuppression is administered to avoid the transplanted organ being rejected by the recipient immune system. However, this lowers patients own immune response and makes the transplant recipient susceptible to opportunistic infections. The commonly used immunosuppressive agents postkidney transplant include steroids, tacrolimus, and mycophenolate mofetil (MMF). All immunosuppressive medicines lower the immune response and make transplant recipient vulnerable to CMV infections. However, MMF is considered most important agent to increase the likelihood of CMV disease by inhibiting proliferation of both *T* and *B* lymphocytes cells suppressing antibody formation and immune response. This case highlights the complex presentation and management of opportunistic infections in recipients of kidney transplants.

Immunosuppressed patients effected with CMV infection commonly present with GI findings. Clinical diagnosis of CMV viremia is difficult given its nonspecific presentation superimposed upon an already complex clinical picture involving ESRD, concurrent comorbidities and postoperative sequelae. Laboratory analysis via CMV PCR is standard in diagnostic work up, as it reliably assesses both viral presence and load to diagnose and track disease progression, respectively [5]. Once diagnosed, management is dependent on symptoms and disease severity. Asymptomatic cases of CMV infection are approached with the goal of mitigating progression to tissue-invasive disease. Medical management involves decreasing immunosuppressive therapy and utilizing antivirals, such as ganciclovir, to effectively decrease viral load [4, 6].

Patients with life-threatening CMV disease are treated with full-dose ganciclovir at 5 mg/kg. Ganciclovir has proven to be effective in viral load reduction and ultimate management of severe CMV viremia in immunocompromised patients [7–9]. Milder cases of CMV infection are often appropriately managed by valganciclovir 900 mg PO twice daily; however, patients with GI tract manifestations may need alterations to their pharmacological strategy. CMV-associated GI disease, as seen in this case, may have jeopardized the patient's ability to maximally absorb and metabolize ganciclovir in the GI tract. The switch from oral to intravenous regimens was adopted to improve bioavailability and tissue penetration guided by patient's general state and oral intake. Also, concurrent risk factors associated with advanced recipient age may have contributed to relative vulnerabilities of CMV reactivation in the post-transplant period [2]. Viral resistance to administered drug is contentious issue which has been less frequently addressed by the literature. However, sequencing to detect mutations can help to identify drug resistant CMV and alternative therapeutic options can be administered [10]. The most common mutations linked to CMV drug resistance are identified in the UL97 gene (H520Q, C592G, A594V, L595S, C603W). This reduces the ganciclovir phosphorylation required for antiviral activity. The alternatives include administration of CMV immunoglobulins (CMVIG) and viral UL97 kinase inhibitors (Maribavir) [11].

The two most common methodologies adopted in the clinical practice for management of CMV in post-transplant renal patients are (1) prophylaxis and (2) pre-emptive treatment. Prophylaxis involves administration of predetermined duration of antiviral medicines based on CMV exposure in the donor and the recipient. High-risk recipients (no previous CMV exposure [donor CMV IgM, IgG will receive prophylaxis for a longer period (6 months) versus transplant recipients with low risk [previous CMV exposure, CMV IgG positive] 3 months). The prophylaxis should be immediately started post-transplant to avoid complication, resulting from viral replication. The recommended course duration is variable between 3 to 6 months [12]. Valganciclovir has been adopted in routine prophylaxis protocols for management of postkidney transplant CMV prophylaxis [12]. The pre-emptive strategy involves regular monitoring of CMV levels in serum of transplant recipients. The objective is to start treatment when serum levels reach a threshold which identifies significant viral load replication before development of symptoms [13].

## 4. Conclusion

CMV viremia presents a significant risk with increased morbidity to transplant recipients. The effective care constitutes early recognition, start of antiviral therapy with monitoring of CMV quantitative levels in patient's serum with sustained reduction in viral load. Ultimately, this is achieved with timely diagnosis and close observation of disease course using a multidisciplinary approach [14–16].

## Data Availability

Data is sharing not applicable to this article as no datasets were generated or analyzed during the current study.
